# Innovative Techniques Associated with Traditional Abdominal Surgery in Complex Pediatric Cases: A Tertiary Center Experience

**DOI:** 10.3390/children8100898

**Published:** 2021-10-09

**Authors:** Rebecca Pulvirenti, Costanza Tognon, Silvia Bisoffi, Filippo Ghidini, Federica De Corti, Francesco Fascetti Leon, Luca Maria Antoniello, Piergiorgio Gamba

**Affiliations:** 1 Pediatric Surgery Unit, Women’s and Children’s Health Department, University Hospital of Padua, 35128 Padua, Italy; silvia.bisoffi@aopd.veneto.it (S.B.); filippo.ghidini@aopd.veneto.it (F.G.); federica.decorti@aopd.veneto.it (F.D.C.); francesco.fascettileon@aopd.veneto.it (F.F.L.); lucamaria.antoniello@aopd.veneto.it (L.M.A.); piergiorgio.gamba@unipd.it (P.G.); 2 Anesthesiology Pediatric Unit, Women’s and Children’s Health Department, University Hospital of Padua, 35128 Padua, Italy; costanza.tognon@aopd.veneto.it

**Keywords:** pediatric surgery, endoscopy, robotic-assisted surgery, new technologies

## Abstract

Pediatric abdominal surgery is constantly evolving, alongside the advent of new surgical technologies. A combined use of new tools and traditional surgical approaches can be useful in the management of complex cases, allowing less invasive procedures and sometimes even avoiding multiple interventions. This combination of techniques has implications even from the anesthetic point of view, especially in post-operative pain control. Thereby, tertiary level centres, including highly-specialized professionals and advanced equipment, can maximize the effectiveness of treatments to improve the final outcomes. Our paper aims to present some possible combinations of techniques recently used at our institution to provide a one-session, minimally invasive treatment within different areas of abdominal surgery.

## 1. Introduction

Pediatric abdominal surgery is constantly evolving. The introduction of new surgical techniques and new therapeutic approaches is playing a key role in the treatment of complex cases, especially when traditional surgery alone would not be able to provide the finest outcome. The advent of surgical technologies that can be combined with traditional techniques allow us to achieve higher survival rates and a better quality of life.

Moreover, a growing interest in national and international multidisciplinary networks combining experts from different specialties is helping to define more complete therapeutic approaches.

As a result, an ever-increasing combination of technologies and approaches is resulting in new therapeutic strategies, allowing surgeons to reach the best outcome possible.

Despite many techniques being well-known and daily-used in the adult population, they are rarely performed in the pediatric population and require careful patient selection. Examples involve robotic-assisted surgery, brachytherapy, MicroWave ablation and fluorescence image-guided surgery.

Referring to robotic surgery, the Da Vinci^®^ Xi system obtained the Food and Drug Administration’s approval in July 2000, and since then has gained popularity within different surgical specialties. In the adult population, it is a daily used technology, especially for urologic and gynecologic interventions, and is increasingly also being used for abdominal surgery. The pediatric population greatly differs in terms of size, physiology and pathology, resulting in obstacles for robotic-assisted surgery. At present, the main limitations refer to the dimensions of trocars and surgical instruments that are not compatible with the size of infants and small children, and the cost-effectiveness of the procedures due to the intrinsic epidemiology of the pediatric diseases [1]. Despite these technical difficulties, robotic-assisted surgery is gaining popularity, especially in the urologic field. In recent years, robotic-assisted pyeloplasty became the technique of choice in adolescents with uretero-pelvic junction stenosis [2], and the European Association of Urology—European Society for Pediatric Urology (EAU–ESPU) guidelines address robotic surgery for pyeloplasty and ureteral reimplantation procedures.

On the other side, brachytherapy and MicroWave ablation are still scarcely used in the pediatric oncologic field, requiring not only a proper patient selection but also sophisticated equipment and the presence of a sub-specialty multidisciplinary team.

Finally, there is near-infrared fluorescence, a fairly new technique for a fluorescence image-guided surgery [3].

Considering that the number of pediatric patients requiring such treatments is quite low, it is difficult to standardize them; in this vision, we report some surgical strategies we applied at our center to treat difficult cases in which the combination of different techniques improved the outcome.

## 2. Endoscopy and Robotic-Assisted Abdominal Surgery

In the last three decades, the number of pediatric endoscopic procedures has increased alongside the therapeutic options possible with this technique and the development of pediatric-adapted instruments [4]. Besides isolated diagnostic or therapeutic endoscopy, the association of this technique with laparoscopic surgery is gaining popularity in abdominal surgeries. In this regard, simultaneous Nissen laparoscopic fundoplication and percutaneous endoscopic gastrostomy (PEG) [5] is one of the main associations performed at our center, mainly in neurologically impaired children [6]. Nissen fundoplication is the most common surgical approach for gastro-esophageal reflux, consisting of a posterior 360 degrees fundoplication, and aiming to re-establish proper esophagogastric junction [7,8]. The mini-invasiveness of this combined procedure favors an uneventful postoperative course, which becomes fundamental in patients with higher perioperative risks, both surgical and anesthetic. Moreover, neurologically impaired children often present a deviation of the spinal column that makes the laparo-assisted approach the safest choice for the gastrostomy positioning. In line with the literature reports [9,10], this approach is safe and becomes crucial in some difficult cases where anatomy might vary due to the underlying disease.

Over the last ten years, our center has adopted the mini-invasive technique as the preferred approach for neurologically-impaired children (after multidisciplinary discussion patients were elected for PEG, laparo-assisted gastrostomy or PEG-combined laparoscopic Nissen fundoplication). From 2008 to 2018, a total of 127 patients underwent a gastrostomy fashioning; 91% of them underwent a Gauderer–Ponsky pull-technique percutaneous endoscopic gastrostomy [11], while the remaining 9% underwent a laparoscopic-assisted PEG fashioning or a Stamm procedure (open gastrostomy) [5]. Gauderer–Ponsky gastrostomy is commonly used, as general anesthesia is not usually required, and because this approach is feasible even in patients with severe musculoskeletal deformities. The technique involves a retrograde passage of the gastrostomy tube through the patient’s mouth, esophagus, gastric and abdominal walls under endoscopic guidance [11]. In the same time period, 18 patients were treated with a simultaneous Nissen laparoscopic fundoplication and gastrostomy fashioning, 5 with an open approach and 13 with a laparoscopic one.

The mean time for gastrostomy fashioning was 23 min. When combined with Nissen fundoplication, the mean operative time was 180 min. The mean postoperative length of stay was five days, mainly due to progressive implementation of enteral nutrition. Short-term minor complications (surgical site infection/granuloma, feeding difficulties, etc.) were observed in 30% of the patients, while short-term major complications (respiratory infections, malfunctioning or displacement of the device, etc.) were detected in 9% of the patients [6].

In 2018, we started a robotic surgery program with the Da Vinci^®^ Xi System and decided to insert Nissen fundoplication, with or without gastrostomy fashioning, in the procedures list for robotic-assisted surgery training. The decision relapsed on this procedure due to the comparable results with the laparoscopic fundoplication reported in literature [12,13]. Furthermore, the use of robotic surgery for Nissen fundoplication might facilitate the surgeon’s work in neurologically impaired children with musculoskeletal malformation. At present, we have performed nine robotic-assisted Nissen fundoplication procedures, four of which were associated with a gastrostomy placement. The mean console time was 144 min with a mean docking time of 13 min. No conversion to open surgery was needed and no major complications occurred. The mean postoperative length of hospital stay was six days.

This combined laparo-endoscopic approach comes with different advantages. The robotic system permits better dexterity of movements thanks to its seven degrees of freedom for instruments [14]; also, the feasibility of the procedure is augmented in patients with a high degree of spinal column deformity, reducing the conversion rate to open surgery. Moreover, during this surgery, the endoscope is used for both the PEG fashioning and to calibrate the diameter of the fundoplication itself. By leaving the instrument through the cardial junction, we assume an adequate esophago-gastric transit can be provided.

A combined use of robotic surgery and endoscopy leads to advantages even from the point of view of anesthesia. From one side, the mean operative time to perform a PEG is shorter compared to both laparoscopic and open gastrostomy [15]; on the other side, robotic surgery grants the maintenance of a stable intra-abdominal pressure. A shorter operative time is advisable in these patients as they frequently present with higher perioperative risks [16].

Additionally, the use of mini-invasive surgical techniques might determine an easier management of post-operative pain [17]. In this regard, besides general anesthesia, different loco-regional blockades are intra-operatively performed, depending on the type of surgery [18]. The most commonly used regional blockades in the case of laparoscopic abdominal surgery are the paravertebral, the transverse abdominis plane (TAP), the erector spinae plane (ESP) and the quadratus lumborum (QL). Local anesthetics used at our center are mainly ropivacaine 0.2% or levobupivacaine 0.125 to 0.175%. Local anesthesia is usually combined with intra-operative opioids and post-operative intravenous medication, mostly acetaminophen, non-steroidal anti-inflammatory drugs and opioid derivates.

## 3. Endoscopy and Abdominal Surgery

A combined use of endoscopy and traditional abdominal surgery is not generally needed apart from the above-mentioned indication. The main limitation to this combined approach is the need for a highly-trained pediatric endoscopist [19,20], a professional figure available mainly in tertiary-care centres. The following cases represent surgical challenges in which a combined approach was extremely helpful in minimizing the invasiveness of the surgical procedure.

The first case was an eight year old boy with short bowel syndrome due to total colonic agangliosis and home parenteral nutrition support. The patient referred a monthly history of increased hematic losses from the distal ileostomy and recurrent anemia. An exploratory laparotomy and ostomy re-do were planned. Intraoperatively, an ileoscopy was performed, which allowed the identification of the ulcerated area. Thereby it was possible to minimize the bowel resection to the affected area and preserve the healthy tissue. Moreover, endoscopy was used to redirect the already on-site JPEG. In line with recent literature reports, endoscopy resulted in an effective diagnostic and therapeutic tool in the treatment of anastomotic ulcers [21].

Another case was an eleven year old girl admitted for a trichobezoar; the mass occupied from the distal third of the esophagus to the proximal third of the duodenum. Endoscopic foreign body removal is widely used and presents a high success rate. However, foreign body dimension, location and impaction time have been described as risk factors predicting conversion to open surgery. [22] A first removal attempt through gastroscopy was made, but proved ineffective. Subsequently, a laparotomy and a gastrotomy were performed. Despite the removal of the gastric part of the mass, the trichobezoar distal extension was such as to require a duodenotomy. In order to avoid such an invasive procedure, a duodenoscopy, starting from the gastrotomy, and the removal of the remaining part of the trichobezoar through an endoscopic grasper were performed.

**Figure 1:**
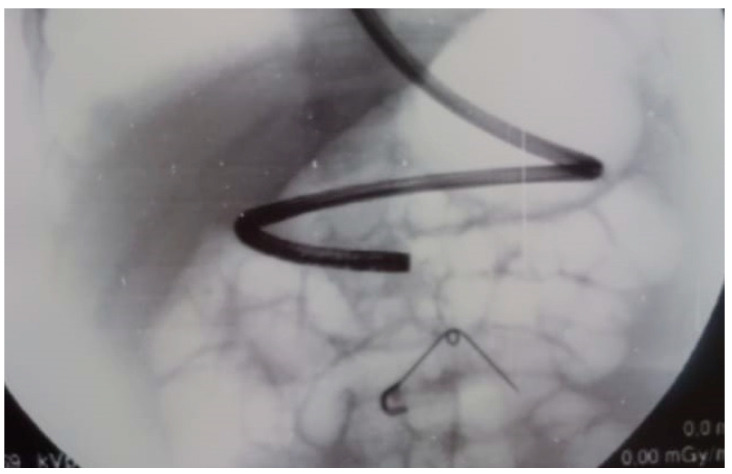
Fluoroscopic guidance of esophagogastroduodenoscopy during the removal of a safety pin.

**Figure 2:**
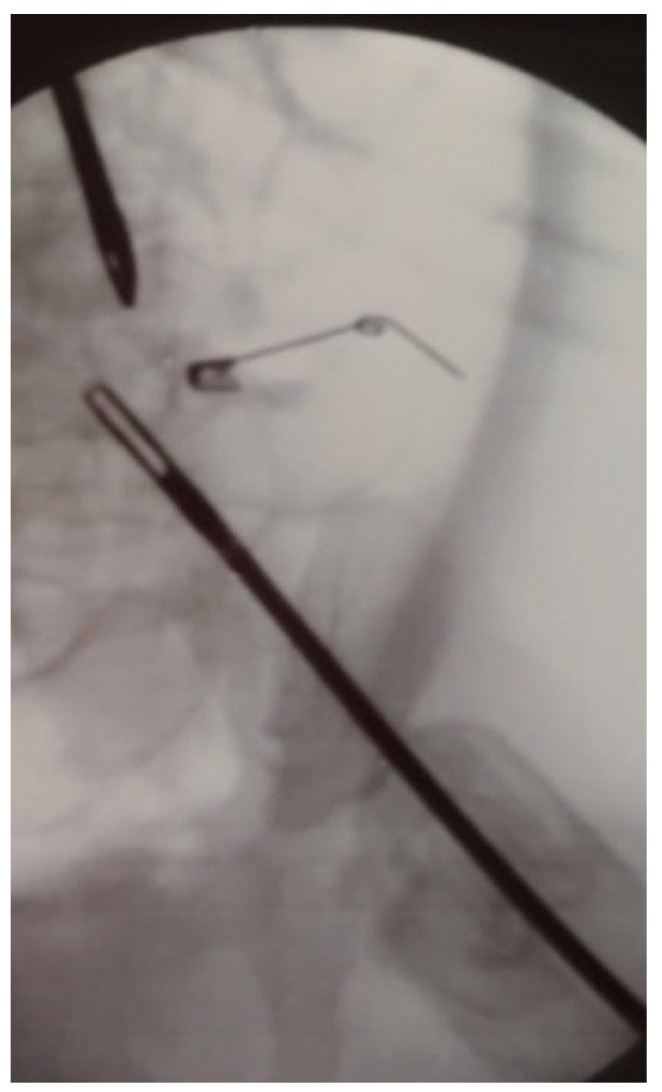
Fluoroscopic guidance of abdominal laparoscopy during the removal of a safety pin.

Finally, there is the case of a nine month old boy admitted for hematemesis. Thoraco-abdominal x-ray revealed a safety pin in the epi-mesogastric region, without signs of pneumoperitoneum or pneumomediastinum. An esophagogastroduodenoscopy first and, subsequently, an exploratory laparoscopy were performed, but no foreign body was found. Lastly, the triangulation obtained with the use of laparoscopic and endoscopic instruments, under fluoroscopic guide, allowed localization of the safety pin at the splenic flexure (Figure 1 and Figure 2). Laparoscopy avoided complete colon insufflation and was used to control the safe, minimally-invasive extraction of the foreign body through colonoscopy [23].

These examples highlight how the combination of different techniques might help in solving situations otherwise requiring very invasive procedures.

## 4. Endoscopic Retrograde Cholangiopancreatography (ERCP) and Laparoscopic Abdominal Surgery

The “rendezvous” procedure [24] is a one-session therapeutic approach to cholecysto-choledocolitiasis, still poorly used in the pediatric population. Besides the rarity of cholecysto-choledocolitiasis among children, the lack of both pediatric-sized instruments and trained pediatric endoscopic teams makes it a safe and effective procedure only in high specialized centers [25].

This technique foresees a simultaneous laparoscopic cholecystectomy, ERCP and endoscopic Oddi’s sphincterotomy.

A peculiarity of this procedure is that it does not require the opening of the common bile duct and a postoperative ERCP.

The main benefits involve a shorter length of stay, the requirement of a single anesthesia and a lower rate of post-ERCP pancreatitis; moreover, in the case of endoscopic stone extraction failure, surgical exploration can be carried out during the same intervention [26].

Etiology of post-ERCP pancreatitis, reported with an incidence between 1 and 14% [27], seems to be related to the volume and the high pressure of the contrast medium injected in the common bile duct [28]. During “rendezvous”, the anterograde injection of the contrast medium by the surgeon avoids its passage into the pancreatic duct and the relative pancreatic hyperpressure, diminishing the risk of pancreatitis development [29].

In addition to pancreatitis, perioperative complications involve bleeding and perforation; a lower incidence of these complication has been described when compared with the two-step procedure [30].

A further advantage of this combined approach is that the ERCP might prove easier to perform, as the surgeon can help the endoscopist by performing an anterograde cholangiography and eventually place a guidewire through the papilla [29]. This may also result in a shorter endoscopic time.

Studies in the adult population defined the rendezvous as an effective procedure for cholecysto-choledocolitiasis, with a common bile duct clearance of 95–100% [31].

**Figure 3:**
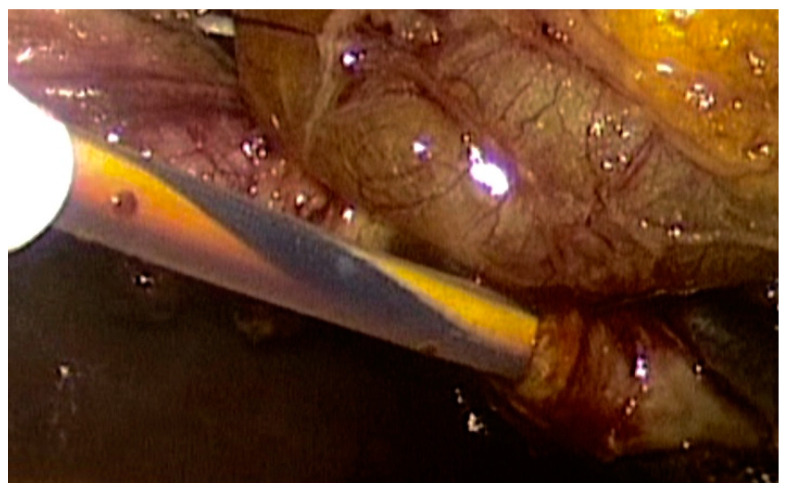
Cannulation of the cystic duct.

To date, we have performed a laparoendoscopic rendezvous (LERV) in three patients affected by symptomatic cholecysto-choledocolitiasis. In all cases, magnetic resonance cholangiopancreatography highlighted the presence of gallstones in the gallbladder and the biliary tree, associated with a dilated common bile duct. The LERV procedure was indicated and performed as described by La Barba et al. [32]. After sphincterotomy, a Dormia basket was introduced into the common bile duct to clean the biliary tree and an outflow of gallstones and biliary sludge was observed (Figure 3) [25]. No perioperative complications occurred and all patients were discharged within seven days.

In our experience, the LERV procedure provides a safe option to treat cholecysto-choledocolitiasis, avoiding multiple interventions and minimizing the length of hospitalization.

## 5. Near-Infrared Fluorescence and Robotic-Assisted Abdominal Surgery

Robotic surgery in pediatric cases largely varies in terms of indications and anatomical targets. This peculiar feature requires the pediatric surgeons to be familiar with most of the instrumentation available for this surgery. Image enhanced surgery has been advocated as the future route to safe dissection. Unfortunately, this technology is far from the routine application [33]. Other tools are currently applied in our practice when a robotic approach is indicated. Near-infrared fluorescence (NIRF) in the adult population is a common application for fluorescence image-guided surgery, and can play a key role for intra-operative decision making.

Referring to abdominal surgery, its main applications involve: recognition of vascular and extrahepatic biliary anatomy, assessment of bowel perfusion before anastomosis and identification of lymphatic draining [34]. Moreover, along with intraoperative ultrasonography, NIRF can be used to confirm safe edges in case of surgical treatment of masses. NIRF mechanism of action relies on a dye, the indocyanine green, which can be injected intravenously and produce fluorescence once excited with a specific wavelength (820 nm) [35]. It is then excreted in the bile [34]. New robotic systems, such as the Da Vinci^®^ Xi, are integrated with the fluorescence imaging [3].

**Figure 4:**
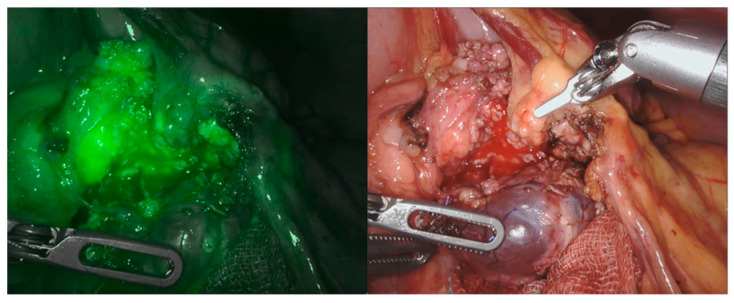
Dissection of a pancreatic cystadenoma, vision with and without NIRF.

**Figure 5:**
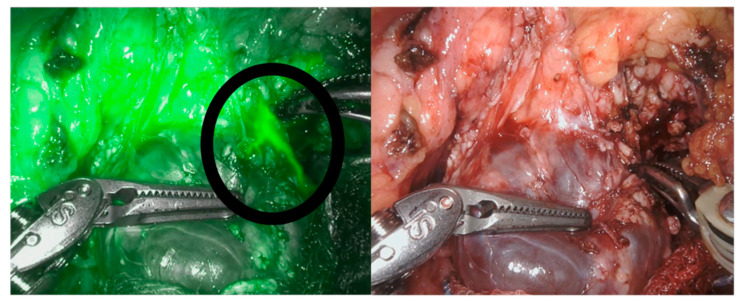
Pancreatic leak visible through NIRF application (circle).

**Figure 6:**
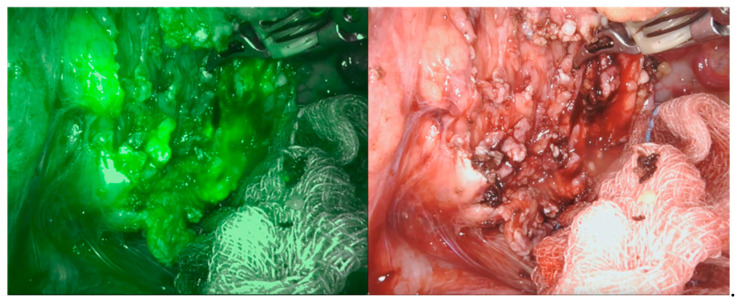
NIRF application during the reconstructive phase.

We recently applied this technology as a supplemental tool for a safe dissection in cases of pancreatic benign masses (in particular, two cases of cystadenoma and two cases of pancreatic cysts). The NIRF application allowed us to properly define the cystic walls (Figure 4) and was also somehow helpful in the reconstructive phase to identify the pancreatic ducts (Figure 5 and Figure 6). Nonetheless, NIRF is not, by any extent, designed to detect leakage of pancreatic secretion, and its usage did not prevent the formation of pancreatic fistula and/or pseudocyst.

We also benefited from NIRF application in a case of combined splenectomy and cholecystectomy for hereditary spherocytosis. In this case, thanks to indocyanine biliary excretion, we were able to identify both vascular anatomy and the cystic duct. After injecting the dye, we were first able to define the splenic vessels and perform a safe robot-assisted splenectomy; once splenectomy was finalised, we used the dye excretion to better identify the cystic duct and perform a more precise dissection for cholecystectomy.

## 6. Intraoperative Ultrasonography and Robotic-Assisted Surgery

A further combination of image-guidance is the application of intraoperative ultrasonography; this is eased by the DaVinci^®^ Xi processor, which allows the visualization of scan images live in the visor during tissue manipulation. The use of real time intraoperative ultrasound has been reported as a useful tool to supply the lack of tactile feedback inherent to laparoscopic or robotic-assisted surgery [36]. In adult patients, it is increasingly being used, as it not only provides a direct vision of the tumor extent on the day of surgery, but also enables the evaluation of tumor mobility during surgical excision [37]. Its main applications in abdominal surgery involve kidney and liver resections. No published data on its effectiveness during robotic-assisted surgery in the pediatric population are available.

**Figure 7:**
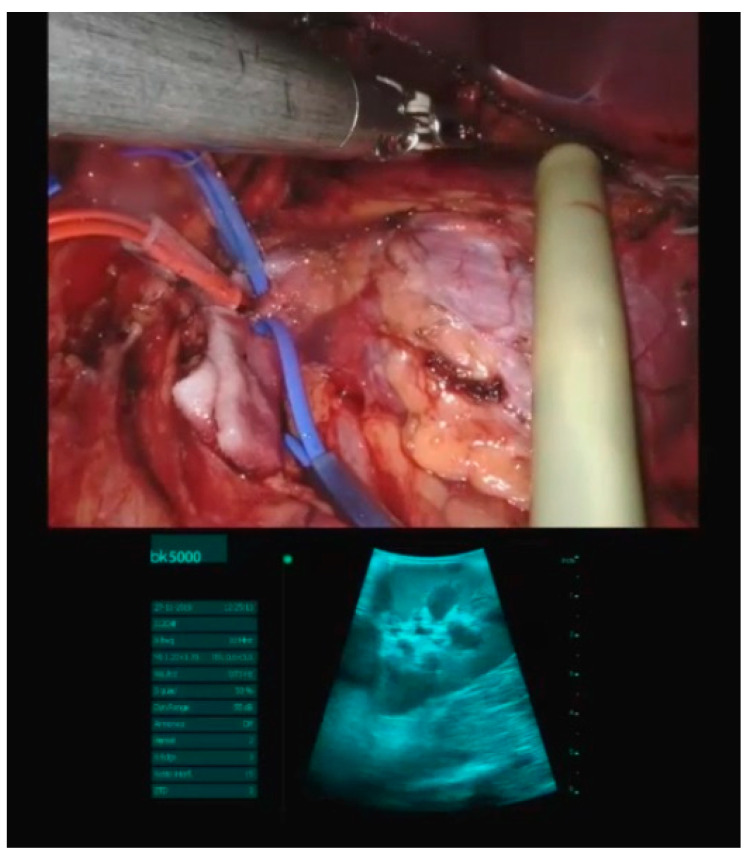
Intraoperative ultrasound during robotic-assisted surgery.

Intraoperative ultrasonography has been applied in our practice for kidney and liver tumors. In a hepatic hamartoma case, we started to isolate the lesion, and by the use of intraoperative ultrasound we were able to better define the edges for an appropriate segmentectomy, preserving hepatic tissue and at the same time performing a radical exeresis of the lesion. Also, in a kidney angiolipoma case we used intraoperative ultrasonography to free the healthy tissue from the disease, maintaining a good renal parenchyma (Figure 7).

## 7. Brachytherapy and Abdominal Surgery

Nowadays, the surgical approach to locally invasive tumors in children should rely on avoiding mutilating interventions. The combination of different strategies, including neoadjuvant chemotherapy, surgery and radiotherapy, is crucial for the treatment of these diseases, especially genitourinary rhabdomyosarcoma, that present extended local invasiveness at diagnosis [38].

To achieve local control in these cases, radiotherapy is as effective as surgery but might increase the risk of local and systemic adverse events through the irradiation of the perineal region [39]. Recently, interstitial brachytherapy has been introduced. This new technique allows the delivery of high doses of radiations directly to target areas, which reduces dispersion and, consequently, side effects [40].

For this reason, the introduction of brachytherapy might change the course of the treatment of genitourinary rhabdomyosarcoma. This new strategy consists of a conservative surgery for the removal of tumor residuals after neoadjuvant chemotherapy and in the placement of trans-perineal tubes during the same surgical intervention. In addition to that, bilateral oophoropexy might be considered in order to preserve ovarian function. The administration of radiotherapy through trans-perineal wires can start after a few days [41].

However, this innovative approach is still presenting several issues regarding patient selection, the different medical figures with multidisciplinary skills required for management, such as oncologists, surgeons, anesthesiologists and radiotherapists, and long-term effects on bladder function [42]. Moreover, it is relevant to underline that the use of sophisticated equipment makes it highly resource-based and available only in tertiary-care centers [40].

Despite the recent introduction, published case-series described a five-year survival rate higher than 90% after this innovative local treatment that combines surgery and brachytherapy [43]. Finally, this new strategy, by granting a higher quality of life [44], might become the leading approach for the local treatment of genitourinary rhabdomyosarcoma in children.

Three patients recently underwent the combined local treatment for genitourinary rhabdomyosarcoma at our institution. All of them underwent conservative surgery followed by brachytherapy and adjuvant chemotherapy; in all three cases the position of tubes was defined together by the surgeon and the radiotherapy oncologist based on the study of the preoperative imaging and the intraoperative appearance of the residuals. For these reasons, four, five and six tubes were placed in the three patients, respectively, in order to give the best opportunity for volume irradiation. After multidisciplinary discussion, an epidural catheter was intra-operatively placed in two patients to ensure proper pain control and increase the tolerance to the devices; the catheter was left in place for 72 h and a combination of chirocaine and fentanyl was continuously infused. Postoperatively, epidural analgesia was associated with multi-agent intravenous medication, mostly acetaminophen at fixed hours, non-steroidal anti-inflammatory drugs and opioid derivates as a rescue option. In the third patient, postoperative pain was controlled with a continuous infusion of morphine at 0.2–0.3 mg/kg/h; vital sign checks and focused neurological exams were regularly performed. All patients remained hospitalized for a couple of weeks, during which two daily brachytherapy sessions were performed. Each brachytherapy session required a sedation, and thus parenteral nutrition support was provided to ensure correct fasting before each anesthesia. Brachytherapy was followed by adjuvant chemotherapy in all cases. None of them presented significant post-voiding after four weeks and the epicystostomic catheter was removed. Our recent experience underlines how the association of surgery and brachytherapy allows the treatment to be tailored to the patient’s and the tumor’s characteristics.

## 8. Microwave Ablation and Abdominal Surgery

MicroWave Ablation (MWA) is a minimally invasive technique for the treatment of small neoplastic lesions, especially of the liver, kidney, bone and lung.

A needle-like device connected to a generator creates an electromagnetic field that delivers heat-energy directly in the target area, causing tissue necrosis [45].

Compared to radiofrequency ablation, MWA requires less time to determine tissue heating while reaching of the target temperature more quickly; a shorter procedure time is particularly important in children as it reduces the anesthesia time and the need for anesthetics administration [46].

Hepatic lesions are the main target of MWA in the pediatric population, and they can be accessed through different approaches: percutaneous, laparoscopic and endoscopic. Few studies comparing the effectiveness of the different approaches are available, preferring the laparoscopic one when treating lesions next to high-risk areas or subcapsular tumors [47].

An ultrasound guidance is needed during the whole procedure, either percutaneous or laparoscopic-assisted.

A complete lesion necrosis, corresponding to surgical excision, requires a 5 mm enlarged ablation beyond the lesion’s margins; residual liver volume is one of the main predictive factors for the development of severe hepatic dysfunction after the procedure.

Possible complications of this technique involve fever, skin burn, abdominal pain, hepatic dysfunction, pleural effusion and intraperitoneal hemorrhage [48].

We recently used this approach to treat a case of liver recurrence of pancreatoblastoma, unresponsive to thermoablation and chemotherapy. Metastatic lesions were defined through laparoscopy and lesion-targeted ultrasound-guided MWA was performed. Complete ultrasound-guided MWA of the main recurrence and two additional subcapsular lesions was assessed. Laparoscopy allowed the exclusion of any extra-hepatic recurrence. During follow-up, the patient developed further retroperitoneal recurrence of disease, but there was no evidence of liver metastatic recurrence after more than twelve months [49]. Literature reports on MWA for relapsed hepatoblastoma in children define a progression-free survival and an overall survival of 9 and 12 months, respectively [48].

## 9. Conclusions

The development of new therapeutic approaches and the spread of these techniques in the pediatric field has assumed ever greater significance in the last years.

At the same time, a multidisciplinary team approach allows a complete therapeutic plan to be set up, especially for complex patients.

Combining techniques belonging to the same or to different sub-specialties might help in diminishing the invasiveness of a surgical procedure and, simultaneously, increase the chances of a good outcome, sometimes even avoiding multiple interventions. Advantages of these therapeutic approaches are remarkable, even from the anesthesia point of view. Patients treated with a one-session surgery require a single anesthesia and, because of the mini-invasiveness of the procedure, an easier management of the post-operative pain.

The availability of advanced equipment and highly-specialized healthcare professionals in a tertiary level centre enables the implementation of increasingly effective therapeutic processes that spread alongside the evolution of medical care.
